# Sensitivity and identifiability analysis of COVID-19 pandemic models

**DOI:** 10.18699/VJ21.010

**Published:** 2021-02

**Authors:** O.I. Krivorotko, S.I. Kabanikhin, M.I. Sosnovskaya, D.V. Andornaya

**Affiliations:** Institute of Computational Mathematics and Mathematical Geophysics of Siberian Branch of the Russian Academy of Sciences, Novosibirsk, Russia Novosibirsk State University, Novosibirsk, Russia; Institute of Computational Mathematics and Mathematical Geophysics of Siberian Branch of the Russian Academy of Sciences, Novosibirsk, Russia Novosibirsk State University, Novosibirsk, Russia; Novosibirsk State University, Novosibirsk, Russia; Novosibirsk State University, Novosibirsk, Russia

**Keywords:** parameter sensitivity, identifiability, ordinary differential equations, inverse problems, epidemiology, COVID-19, forecasting, Novosibirsk region, чувствительность параметров, идентифицируемость, обыкновенные дифференциальные уравнения, обратные задачи, эпидемиология, COVID-19, прогнозирование, Новосибирская область

## Abstract

The paper presents the results of sensitivity-based identifiability analysis of the COVID-19 pandemic spread models in the Novosibirsk region using the systems of differential equations and mass balance law. The algorithm is built on the sensitivity matrix analysis using the methods of differential and linear algebra. It allows one to determine the parameters that are the least and most sensitive to data changes to build a regularization for solving an identification problem of the most accurate pandemic spread scenarios in the region. The performed analysis has demonstrated that the virus contagiousness is identifiable from the number of daily confirmed, critical and recovery cases. On the other hand, the predicted proportion of the admitted patients who require a ventilator and the mortality rate are determined much less consistently. It has been shown that building a more realistic forecast requires adding additional information about the process such as the number of daily hospital admissions. In our study, the problems of parameter identification using additional information about the number of daily confirmed, critical and mortality cases in the region were reduced to minimizing the corresponding misfit functions. The minimization problem was solved through the differential evolution method that is widely applied for stochastic global optimization. It has been demonstrated that a more general COVID-19 spread compartmental model consisting of seven ordinary differential equations describes the main trend of the spread and is sensitive to the peaks of confirmed cases but does not qualitatively describe small statistical datasets such as the number of daily critical cases or mortality that can lead to errors in forecasting. A more detailed agent-oriented model has been able to capture statistical data with additional noise to build scenarios of COVID-19 spread in the region.

## Abstract (ru)

Разработан алгоритм анализа чувствительности и идентифицируемости математических моделей распространения эпидемии COVID-19 в Новосибирской области, основанных на системах дифференциальных уравнений и законе действующих масс. Основу алгоритма составляет анализ матрицы чувствительности методами дифференциальной и линейной алгебры, показывающей степень зависимости неизвестных параметров моделей от заданных измерений. В результате работы алгоритма выявляются наименее и наиболее чувствительные к измерениям параметры, что способствует построению регуляризующего алгоритма решения задачи идентификации параметров для построения более точных сценариев развития эпидемии в регионе. Анализ чувствительности математических моделей распространения коронавирусной инфекции COVID-19 показал, что параметр контагиозности вируса устойчиво определяется по количеству ежедневно выявляемых заболевших, критических и вылечившихся больных. С другой стороны, прогнозируемая доля госпитализированных больных, находящихся в критическом состоянии и требующих подключения аппарата ИВЛ, а также коэффициент смертности определяются гораздо менее устойчиво. Для построения более реалистичного прогноза необходимо добавить дополнительную информацию о процессе (например, о количестве ежедневных случаев госпитализации). Задачи уточнения идентифицируемых параметров по дополнительной информации о количестве выявленных, критических и смертельных случаев в Новосибирской области были сведены к задачам минимизации соответствующих целевых функционалов. Задача минимизации была решена с помощью метода дифференциальной эволюции, широко используемого в задачах стохастической глобальной оптимизации. Показано, что более общая камерная модель, состоящая из семи обыкновенных дифференциальных уравнений, описывает основную тенденцию распространения коронавирусной инфекции, чувствительна к пикам выявленных случаев, однако некачественно описывает небольшие статистики (количество ежедневных критических, смертельных случаев), что может приводить к ошибочным выводам. Более подробная агентно-ориентированная математическая модель, учитывающая поведение отдельных агентов, позволяет улавливать небольшие шумы в данных и строить сценарии развития распространения эпидемии в регионе.

## Introduction

Many mathematical models in biology (epidemiology, immunology, pharmacokinetics, systems biology), medicine (tomography), physics and chemistry (meteorology, chemical kinetics), as well as sociology are described by systems of differential equations, whether they be ordinary (Kermack, McKendrick, 1927), partial (Habtemariam et al., 2008), or stochastic differential ones (Lee et al., 2020). Coefficients in these equations characterize specific features of simulated processes under given conditions. To build an adequate mathematical model, one needs to refine the coefficients of the equations based on the known parameters of the process and any additional information available about it. For example, when considering epidemiological problems, the parameters such as the infection transmission rate in the region; the critical case rates depending on comorbidities, age, and other demographic factors; the proportion of asymptomatic carriers/latent infection cases, etc., are unknown or approximately derived based on statistical data. These parameters are often sensitive to the measurements prone to errors (rounding, instrument, and human factor errors), which leads to unstable solutions of parameter identification problems

Identifiability analysis of the differential equation systems modeling biological, medical, and physical processes is an important step to undertake before developing computational algorithms (Bellu et al., 2007; Raue et al., 2010, 2014; Miao et al., 2011; Kabanikhin et al., 2016; Voropaeva, Tsgoev, 2019). Aclassification of identifiability types distinguishing between structural identifiability, practical identifiability, and sensitivity analysis is presented in (Krivorotko et al., 2020a). The authors also analyze the systems of ordinary differential equations (ODE) describing epidemiological and immunological processes in terms of practical identifiability and parameter sensitivity to measurement errors.

A detailed review of methods and case studies of structural identifiability analysis in biological problems described by ODE systems may be found in (Miao et al., 2011; Kabanikhin et al., 2016). The model structure being as follows:

**Formula:**
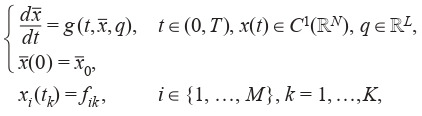
(1)

In the present paper, the analysis of the semi-relative sensitivity of the mathematical models to describe epidemiological and social processes is presented. This approach, proposed in (Adams et al., 2004) for analyzing ODE systems, shows the degree of parameter sensitivity to measurements and identifies lacking/excessive measurements based on a certain reference set of parameters for solving the stated parameter identification problem. Two mathematical models of the spread of the new coronavirus infection caused by the SARS-CoV-2 virus described by ODE systems are used as examples. A regularization algorithm for numerical solution of the parameter identification problem is developed for SEIR compartmental model and agent-based model using the statistical data from public sources. The modeling results and the scenario of COVID-19 spread in the Novosibirsk region are presented.

## Parameter sensitivity analysis in systems of ordinary differential equations

Sensitivity analysis is used for identifiability assessment of the unknown parameters of the model represented by ODE system (1) before developing a numerical solution algorithm for the parameter identification problem. These methods do not require real experimental data, but the number of measurements and their time may be a necessity. Sensitivity analysis for a mathematical model is performed with regard to a set of nominal parameters q*, whose values are taken from the literature or statistical data available.

Sensitivity analysis methods are based on a sensitivity matrix. Assume that t 1 ≤ t 2 ≤…≤ t K are the fixed times of measurementsf ik . Then, the sensitivity matrix coefficients for parameter vector q* are calculated as:

**Formula:**
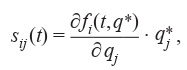
(2)

where, f i , i = 1, …, M , is the i th entry of the measurement function vector, and qj , j = 1, …, L, is the j th entry of the parameter vector

Thus, the sensitivity matrix is determined as follows:

**Equation:**
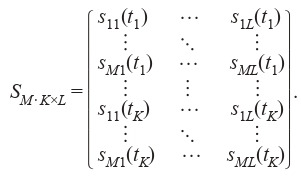
1

The sensitivity matrix is calculated using the conventional sensitivity function:

**Equation:**
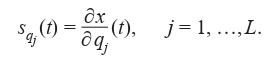
2

When the first equation from (1) is differentiated with respect to qj , each vector function sqj should satisfy the Cauchy problem as follows:

**Formula:**
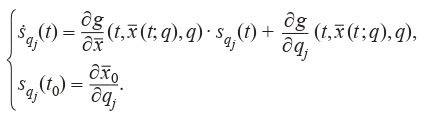
(3)

So, sqj (t) is obtained by numerically solving the Cauchy problem.

First, the assessment is performed for the parameters q, to which the model’s solution is most sensitive. These parameters, in turn, are defined by calculating semi-relative sensitivity. Here, sensitivity is considered a time function on the interval of interest. To obtain a general measure of parameter sensitivity of the solution, a time norm (over space L2) is derived for each state/parameter combination and the obtained scalar quantities are ranked to identify the most sensitive parameters. The lower the value (Equation 3), the less the effect of qk on f i . This general measure will be referred to as semirelative sensitivity

**Equation:**
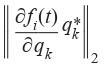
3

The orthogonal method is then used for sensitivity analysis. The idea of the method suggested in (Yao et al., 2003) is to investigate linear dependencies of the columns of sensitivity matrix S. In such a way it will be possible to assess parameter sensitivity to the input data and parameter interdependence at the same time. 

## Sensitivity analysis of COVID-19 spread models

The feature of currently developed COVID-19 spread models is that they analyze the behavior of asymptomatic cases and the effect of the incubation period on the epidemiological situation in the regions. Several open-source suites (Gomez et al., 2020; Tuomisto et al., 2020; Wolfram, 2020) and web services have been developed for modeling COVID-19 spread scenarios:

- on a global scale: https://covid19-scenarios.org/ (University of Basel, Switzerland);
- in Moscow, the Novosibirsk region, and some European countries: https://covid19.biouml.org/ (Institute of Computational Technologies, SB RAS, Novosibirsk);
- in Almaty, the Republic of Kazakhstan: http://covid19.mmay.info/almaty/?fbclid=IwAR20yx7F4MdWRqwUDzripUK29lWAvoyCSkDPafgpj25ummay23e7oFHBdXg.

Two fundamental approaches to epidemic propagation modeling may be distinguished:

- Compartmental approach (top-down modeling). Here, the interaction between the agents within the population grouped by similar attributes (susceptible group, (a)symptomatic carriers, hospital admission cases, critical cases, etc.) is described using the mass balance law within the compartmental model first suggested in 1927 (Kermack, McKendrick, 1927). Agents are distributed in time depending on the assigned transition probabilities between groups such as infection probability, virus contagiousness, mortality rate, etc.
- Agent-based approach (bottom-up modeling) is based on studying the interactions between individuals and their effect on global parameters (e. g. virus contagiousness, mortality rate, severe case probability, etc.). Agent-based models are characterized by random graphs whose arc lengths describe the probabilities of transition to different agent states.

The parameters of transition between groups or agent states are often unknown or broadly defined. For instance, the incubation period of the disease according to WHO data varies from 2 to 14 days, which complicates the analysis of the model and the building of adequate disease spread scenarios.

**Fig. 1.**
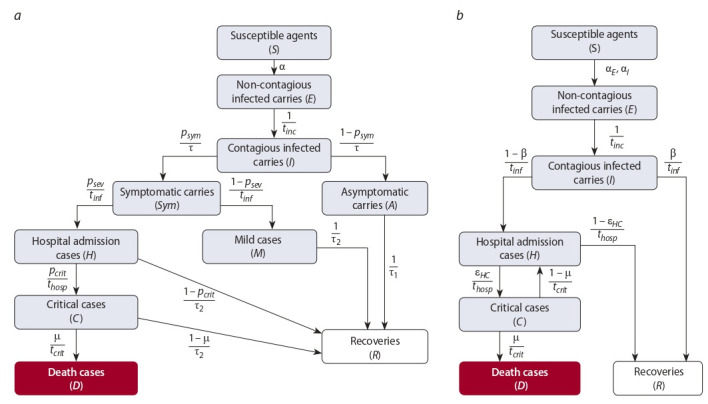
Agent-state diagram in (a) the COVASIM package (Kerr et al., 2020) and (b) the SEIR-HCD model (Unlu et al., 2020).

Let us consider two breakdowns of the population of a particular age (e.g., ages 20–29) into groups. The transition into different agent states in the course of the disease caused by the SARS-CoV-2 virus is presented in Fig. 1. These models do not take into account such factors as sex segregation, annual birth and mortality rates (since the modeling interval of less than a year is analyzed), vaccination, passenger traffic, and comorbidities, which affect the probabilities of transition to different agent states. Our goal here is to demonstrate the correlation between dependences of similar parameters on the same measurements and recommend what parameters can be determined consistently and based on what measurements.

The ODE system (1) describing COVID-19 spread in the population is divided into 10 groups (Kerr et al., 2020) based on the mass balance law and is expressed as follows:

**Formula:**
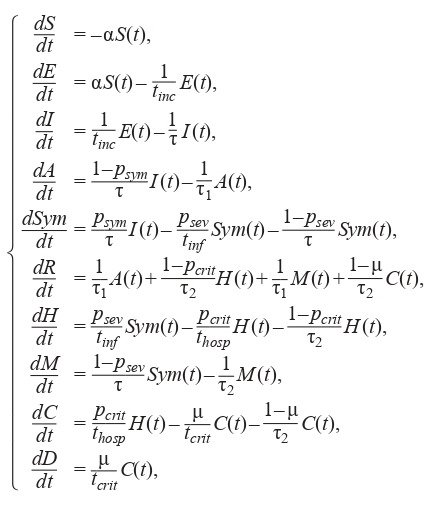
(4)

with the initial conditions:

**Equation:**
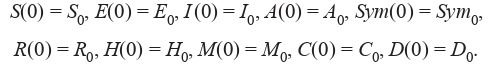
4

Model (4) characterizes a class of agent states for an age group within the agent-based model (see Fig. 1, a).

The equation system for the SEIR-HCD model, where the population is divided into 7 groups (Krivorotko et al., 2020b; Unlu et al., 2020), is composed in a similar fashion:

**Formula:**
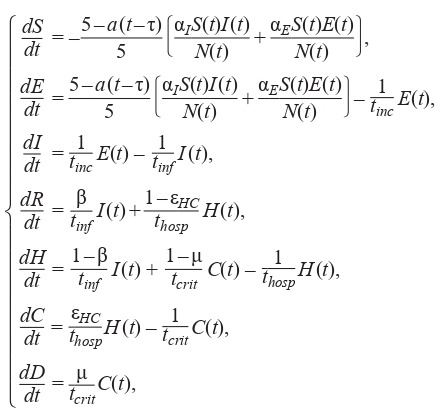
(5)

with the initial conditions:

**Equation:**
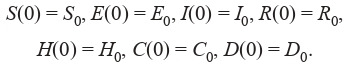
5

Here, S (t) is a susceptible agent at time t, E(t) – a noncontagious infected (not transmitting the virus), I (t) – a contagious infected (transmitting the virus), A(t) – an asymptomatic case, Sym(t) – a symptomatic case, H (t) – a severe case, C (t) – a critical case (requiring a ventilator), M (t) – a mild case, R(t) – a recovered case, and D (t) – a mortality case. The averaged parameters of models (4) and (5) for the Novosibirsk region are presented in Table 1 (Lauer et al., 2020; Verity et al., 2020; Wölfel et al., 2020).

**Table 1.**
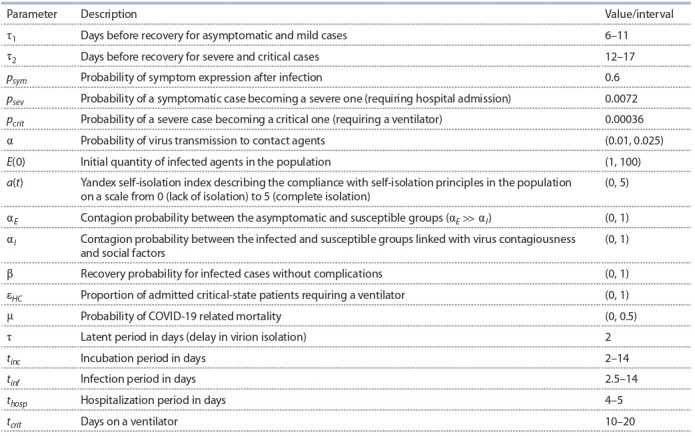
Averaged parameters used in models (4), (5) for the Novosibirsk region (Kerr et al., 2020; Unlu et al., 2020)

Note that the coefficients t –1 inc , t –1 inf , t –1 hosp, t –1 crit, τ –1, τ–1 1 , τ–1 2 at the respective agent states in models (4) and (5) describe the delay in transition between states (Likhoshvai et al., 2004). Consider the following equation (5):

**Equation:**
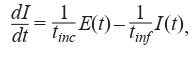
6

where coefficient t –1 inc (in the linear approximation) indicates the delay of t inc days before the transition from non-contagious infected group E(t) to contagious infected group I (t), and coefficient –t –1 inf – that the agent stays in the contagious infected group for the infection period of t inf days

Mathematical model 1 (see the diagram in Fig. 1, a). Assume that additional information on recoveries and deaths on fixed days is available for the mathematical model (4):

**Formula:**
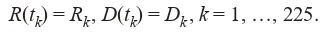
(6)

Here, Rk is the number of recovered agents on day k, Dk is the number of disease-related deaths on day k. 

The model analyzes the semi-relative sensitivity of two unknown parameters, i. e. contagiousness α and initial quantity of asymptomatic cases E(0) in the model (4) to the measurements (6). It will allow us to determine the possibility of consistent identification of the unknown parameters based on the data available for an adequate representation of epidemiological situation in the region. The sensitivities of parameters (qk) = (α, E(0)), k = 1, 2, to measurements ( f i ) = (R, D), i = 1, 2, represented by norm (Equation 7) and sorted in descending order are presented in Table 2. The lower the value (Equation 7), the less the effect of qk on f i .

**Equation:**
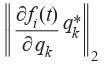
7

**Table 2.**
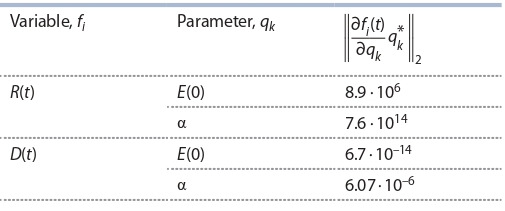
Semi-relative sensitivities of various model (4) states to the parameters sorted in descending order

**Fig. 2.**
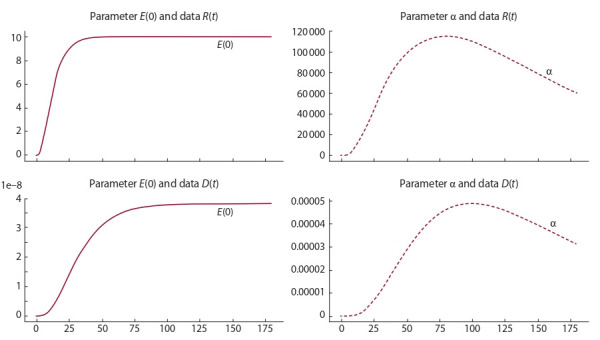
Sensitive function (Equation 7) for model (4) for the period from 12.03.2020 to 09.09.2020 (182 days).

Figure 2 demonstrates how sensitive function (Equation 8) changes in time depending on the parameter. Thus, parameters α and E(0) within the model (4) are less sensitive to variable D (t) and are therefore not identifiable by the mortality data alone. On the other hand, these parameters are sensitive to function R (t), and, as a result, are recovered more consistently based on the recovery data.

**Equation:**
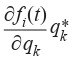
8

**Mathematical model 2** (see the diagram in Fig. 1, b). Let us now investigate the SEIR-HCD mathematical model (5). Assume that additional information on diagnoses, critical cases, and mortality on fixed days is available:

**Formula:**
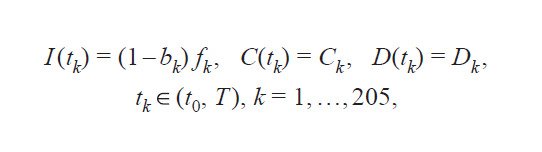
(7)

where b(t) [0, 1] is the proportion of asymptomatic carriers in the diagnoses, f k – the daily number of diagnoses on day k, Ck – the number of critical cases on day k.

Parameters q = (αE, αI, β, εHC, μ, E0)T ℝ6 are considered unknown. To analyze the semi-relative sensitivity of the parameter vector q to measurements (7) within the mathematical model (5), we derive (Equation 8) ( f i ) = (I, C, D), i = 1, 2, 3, (Equation 7) (Table 3). Consistency of identifying parameters β, εHC, and μ as the result of solving the inverse problem barely depends on the available measurements of the number of infected carriersI (t); however, it is not the case for the more sensitive coefficients αE, αI, E0. and analyze the values

**Table 3.**
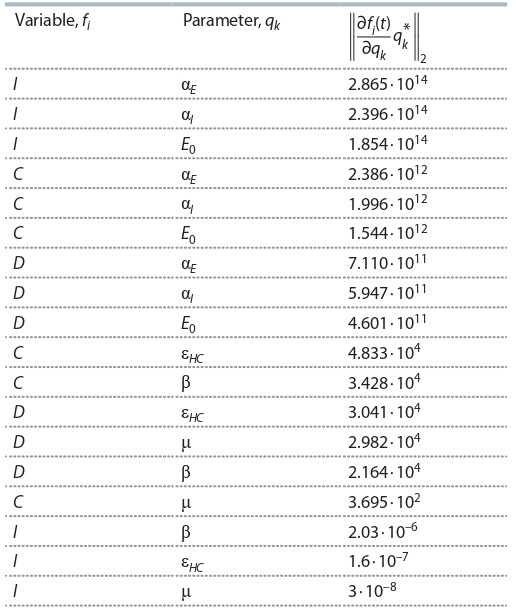
Semi-relative sensitivities of various states of the model (5) to parameters, sorted in descending order

**Fig. 3.**
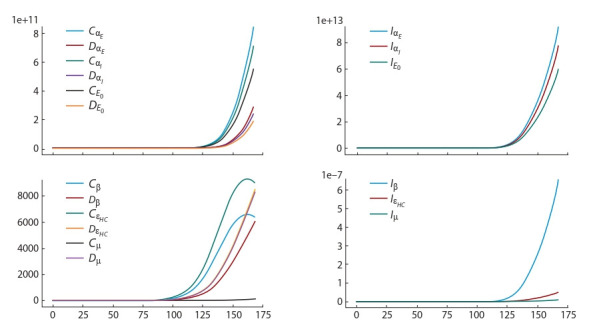
Semi-relative sensitivity function (Equation 7) for the time interval from 15.04.2020 to 01.10.2020 (170 days)

Figure 3 shows how sensitive function (Equation 7) changes in time depending on the parameter. The more the parameter changes in time, the higher its sensitivity to the measurements analyzed, and the more consistently it is identified.

**Fig. 4.**
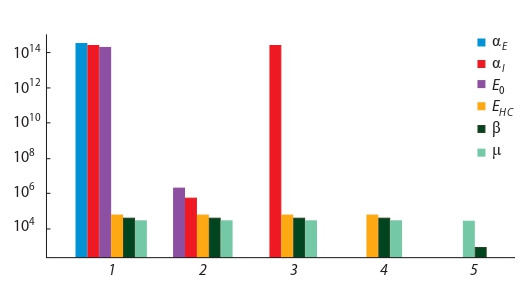
Normalised perpendiculars for each parameter (different colors) at the different iterations (1–5) of the sensitivity-based orthogonal algorithm (5)

In Fig. 4, the results of parameter sensitivity analysis for the model (5) at various iterations of the orthogonal algorithm are presented (see the description of the algorithm in (Krivorotko et al., 2020a)). Iterations of the orthogonal algorithm, whose total number is one less than the dimension of the unknown parameter vector (i. e. the number of columns in the sensitivity matrix), are plotted along the horizontal axis, and the norms of perpendiculars for the obtained transformations of sensitivity matrices – along the vertical axis. It was shown that the contagion between the asymptomatic and susceptible groups αE, the contagion between the infected and susceptible groups αI linked with virus contagiousness and social factors, and the initial quantity of infected carriers and the agents in incubation period E0 turned out to be the more identifiable parameters. The ranking of the parameters obtained via sensitivity analysis for the model (5) from the most sensitive to the least sensitive is as follows: αE, E0, αI, εHC, μ, β.

As a result of identifiability analysis, a conclusion can be made that model parameters αE, E0, and αI are the least sensitive to data variations (errors), i. e. are more identifiable. In other words, these parameters are identified more consistently as a result of solving the inverse problem (5), (7). In turn, parameters εHC, μ, and β are the most sensitive to measurement errors, i. e., less identifiable (and have the lowest values of the norms of perpendiculars in the sensitivity matrix). Hence, the regularization algorithm should be developed to ensure the consistent identification of sensitive parameters.

## Mathematical modeling of COVID-19 spread in the Novosibirsk region

To build a COVID-19 spread model for the Novosibirsk region, the following publicly reported data were used:

- Number of people tested (including the number of diagnoses f and proportion of asymptomatic carriers b(t)), recovered cases (R), and COVID-19 related deaths (D);
- Duration of incubation period t inc , latent period τ, infection period t inf , hospitalization period t hosp , and duration of ventilation t crit ;
- Recovery time for mild τ1 and severe τ2 cases;
- Demographic profiles (population size and its age distribution in the region);
- Average household size (2.6 people) in the Russian Federation in 2019, according to UN data (https://population. un.org/Household/#/countries/840).

Additional information was regularly obtained from the following websites:

- Ministry of Health of the Novosibirsk region: https://zdrav. nso.ru/ (d).
- Federal State Statistics Service of the Novosibirsk region: https://novosibstat.gks.ru/folder/31729 (c).
- Stopcoronavirus website: https://стопкоронавирус.рф (a).
- World Health Organization: https://www.who.int (b).

The modeling was performed taking into account the measures to contain the COVID-19 spread (Table 4).

**Table 4.**
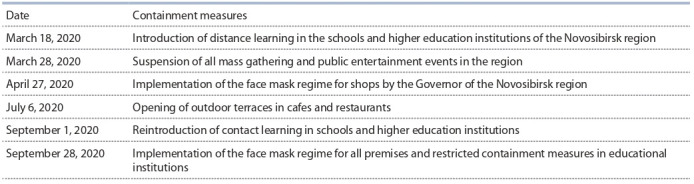
COVID-19 containment measures in the Novosibirsk region to be used in the models (4), (5)

Solutions of inverse problems (4), (6) and (5), (7) were reduced to misfit function minimization (Kabanikhin, 2008):

**Equation:**
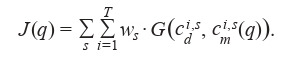
9

Here, s is the statistics used for data comparison (cumulative diagnoses, critical cases, and mortality), ws – the weight coefficient, c i, s d , c i, s m – data (with subscript d ) and model values (with subscript m), T – the modeling interval in days, q – the unknown parameter vector: q1 = (β, E0)T for the inverse problem (4), (6) and q2 = (αE(t), αI(t), β, εHC, μ, E0)T for the inverse problem (5), (7). The absolute norm for computational experiments was set as follows:

**Equation:**
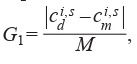
10

where M = max t {c t, s d } was the normalization item; and the standard deviation was

**Equation:**
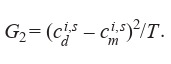
11

Minimization of misfit function J (q) was implemented using the differential evolution method from the SciPy.Optimize Python library. The general algorithm of global minimum search was as follows:

1. Creation of the initial generation {qi } B, i = 1…N..

2. Creation of a new generation: • Mutation: For all qi B three random vectors were selected as follows: v1, v2, v3 B, (vj ≠ qi, j = 1, 2, 3). Mutant vector: v = v1 + F(v2 – v3), F [0, 2]. • Crossover: trial vector u was calculated as follows:

**Equation:**
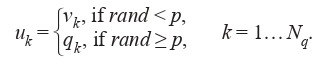
12

3. Selection:

**Equation:**
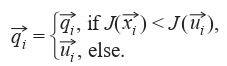
13

**Fig. 5.**
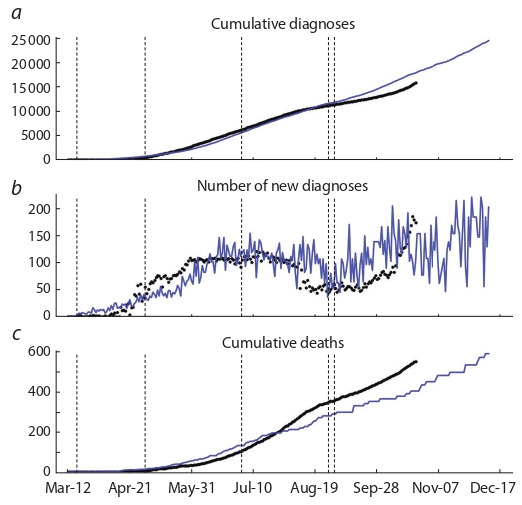
COVID-19 spread model for the Novosibirsk region (solid blue line) using the agent-based approach and statistical data (black dots) with containment measures (vertical dashed lines).

The results of COVID-19 spread modeling in the Novosibirsk region with the forecast up to December 10, 2020, are presented in Fig. 5. The model was built using the agent-based approach relying on the investigation of interactions between individuals and their effect on global parameters. The modeling was performed using Covasim, a simulator for developing stochastic agent-based models. A detailed discussion of the model structure may be found in (Kerr et al., 2020). We also used the statistical data on diagnoses and deaths from March 12 to October 23, 2020. The following misfit function was minimized taking into account the identifiability analysis results for the model (4), (6):

**Equation:**
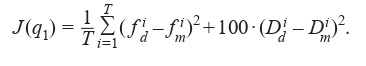
14

Here, f i d, f i m are cumulative diagnoses, and D i d, D i m are cumulative deaths

Modeling results f i m and statisticsf i d of cumulative and daily diagnoses are presented in Fig. 5, a, b. Modeling results D i m and statistics D i d of cumulative COVID-19 related deaths in the Novosibirsk region are presented in Fig. 5, c. Note that the second wave of the epidemic may be observed in the Novosibirsk region in mid-September in both the statistical data and modeling results. Its growth will be insignificant (i. e. it will not exceed 215 new daily diagnoses by mid-December, 2020) due to the introduction of stricter containment measures from October 28.

The inverse problem (5), (7) was reduced to the minimization of the following misfit function (Krivorotko et al., 2020b):

**Equation:**
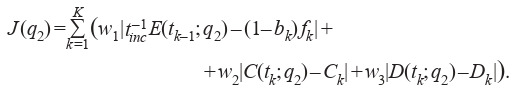
15

Infection rate parameters αE (t) and αI(t) linked to virus contagiousness and varying in time were represented as piecewise constant functions depending on the interventions (see Table 4).

**Fig. 6.**
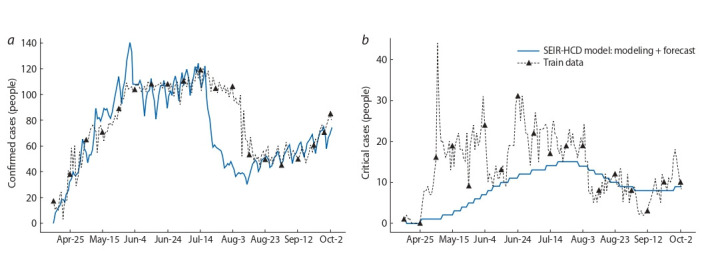
Modeling COVID-19 spread in the Novosibirsk region (solid blue line) from 15.04.2020 to 03.10.2020 and the statistical data (dashed black line) for (a) daily confirmed cases fk and (b) the critical cases Ck requiring a ventilator

Based on the identifiability analysis results for the model (5), (7), more rigid restrictions were imposed for poorly identifiable parameters (see Table 1). The result of solving the inverse problem (5), (7) for the SEIR-HCD model for the period from April 15, 2020, to October 3, 2020, is presented in Fig. 6.

Note that although the rough mathematical model (with ODE system of 7 equations) captures the general trend based on the number of diagnoses (the peak of confirmed cases in the region, see Fig. 6, а), it is still unable to qualitatively describe highly variable statistics (critical cases requiring a ventilator, see Fig. 6, b). Nonsmooth solutions in Fig. 6 result from the use of the Yandex self-isolation index characterized by weekly seasonality. In this case, smoothing would undermine the use of the tool. A more detailed analysis of modeling and forecasting of COVID-19 spread in the Moscow and Novosibirsk regions is presented in (Krivorotko et al., 2020b). This case requires the agent-based approach capable of detailed description of small statistical datasets.

## Conclusion

In the present study, sensitivity-based identifiability analysis has been performed for the COVID-19 pandemic spread models based on systems of differential equations. The algorithm is based on the analysis of the sensitivity matrix using the differential and linear algebra apparatus, which shows the degree of dependence of the unknown model parameters on the given measurements.

The analysis has shown that the virus contagiousness is consistently identified based on the number of new daily diagnoses, critical cases, and recoveries. On the other hand, the predicted proportion of admitted critical state patients requiring a ventilator and the mortality rate are identified much less consistently. It has been demonstrated that developing a more realistic forecast will require additional information about the process such as the number of daily hospital admissions.

The identifiable parameters refinement problems have been reduced to the minimization of the respective misfit functions describing the proximity of the modeling data to the statistics of the diagnoses, critical cases, and mortality in the Novosibirsk region. The use of absolute and quadratic norms as measures of deviation between the data and the modeling results in the minimization procedures has not yielded any significant differences in terms of analyzing the modeling results. It has been shown that a rough compartmental model of seven ODEs describes the general trend of the coronavirus infection propagation, as it is sensitive to peaks of confirmed cases; however, it is unable to qualitatively describe smaller statistics (daily numbers of critical casest k and deaths), which may lead to improper conclusions. Amore detailed mathematical model using the agent-based approach, where a class of agent states is expressed by a system of ten ODEs, will make it possible to describe noisy statistical datasets in more detail and build adequate scenarios of COVID-19 pandemic spread.

## Conflict of interest

The authors declare no conflict of interest.
